# Vaccines against Coronaviruses: The State of the Art

**DOI:** 10.3390/vaccines8020309

**Published:** 2020-06-17

**Authors:** Cristiano Conte, Francesco Sogni, Paola Affanni, Licia Veronesi, Alberto Argentiero, Susanna Esposito

**Affiliations:** 1 Pediatric Clinic, Department of Medicine and Surgery, Pietro Barilla Children’s Hospital, University of Parma, 43126 Parma, Italy; cristianoconte1993@gmail.com (C.C.); francesco.sogni@hotmail.it (F.S.); aargentiero85@gmail.com (A.A.); 2 Department of Medicine and Surgery, University of Parma, 43126 Parma, Italy; paola.affanni@unipr.it (P.A.); licia.veronesi@unipr.it (L.V.)

**Keywords:** coronavirus, MERS, pandemic, SARS, SARS-CoV-2, vaccine candidate

## Abstract

The emerging epidemic caused by the new coronavirus SARS-CoV-2 represents the most important socio-health threat of the 21st century. The high contagiousness of the virus, the strong impact on the health system of the various countries and the absence to date of treatments able to improve the prognosis of the disease make the introduction of a vaccine indispensable, even though there are currently no approved human coronavirus vaccines. The aim of the study is to carry out a review of the medical literature concerning vaccine candidates for the main coronaviruses responsible for human epidemics, including recent advances in the development of a vaccine against COVID-19. This extensive review carried out on the vaccine candidates of the main epidemic coronaviruses of the past has shown that the studies in animal models suggest a high efficacy of potential vaccines in providing protection against viral challenges. Similar human studies have not yet been carried out, as the main trials are aimed at assessing mainly vaccine safety and immunogenicity. Whereas the severe acute respiratory syndrome (SARS-CoV) epidemic ended almost two decades ago and the Middle East respiratory syndrome (MERS-CoV) epidemic is now better controlled, as it is less contagious due to the high lethality of the virus, the current SARS-CoV-2 pandemic represents a problem that is certainly more compelling, which pushes us to accelerate the studies not only for the production of vaccines but also for innovative pharmacological treatments. SARS-CoV-2 vaccines might come too late to affect the first wave of this pandemic, but they might be useful if additional subsequent waves occur or in a post-pandemic perspective in which the virus continues to circulate as a seasonal virus.

## 1. Introduction

The emerging epidemic caused by the new coronavirus SARS-CoV-2 represents the most important socio-health threat of the 21st century [1]. Two other human coronaviruses have been identified in recent years as being responsible for severe lung infections: severe acute respiratory syndrome (SARS-CoV) and Middle East respiratory syndrome (MERS-CoV) coronavirus [2]. The wide spread of the virus on all continents has led the World Health Organization (WHO) to declare a pandemic. As of 27 April 2020, 2,900,053 cases have been registered in 185 countries, causing over 200,000 deaths. The main affected regions are the USA, Europe, the Middle East, Russia and China. The high contagiousness of the virus, the strong impact on the health system of the various countries and the absence to date of treatments able to improve the prognosis of the disease make the introduction of a vaccine indispensable, even though there are currently no approved human coronavirus vaccines. In the meantime and while hoping that this will happen, the most affected regions have adopted socio-economic health measures designed to limit the propagation of SARS-CoV-2, up to the total “lockdown” of some regions. The main research laboratories worldwide are fighting against time to stop the spread of this terrible epidemic, but they are subject to the technical restraints of time for pharmaceutical experimentation. Time frames could be shorter than usual considering the previous lessons from other coronavirus vaccine experimentation.

The aim of the study is to carry out a review of the medical literature concerning vaccine candidates for the main coronaviruses responsible for human epidemics, including recent advances in the development of a vaccine against COVID-19. We performed bibliographic research from 1 January 2000, to 15 May 2020, through mainly the PubMed platform, comparing some information obtained with the WHO, Italian Higher Institute of Health, National Institutes of Health (NIH) and Clinical Trial sites.

## 2. Biology of Coronaviruses

SARS-CoV-2 belongs to the *Coronaviridae* family, subfamily *Orthocoronavirinae*, genus Betacoronavirus, [1] which includes two other known viruses responsible for past epidemics, SARS-CoV (2002) and MERS-CoV (2012) [3], in addition to the human coronaviruses associated with common seasonal respiratory infections (HCoV-229E, HCoV-OC43, HCoV-NL63 and HCoV-HKU1) [4].

Coronaviruses are ubiquitous viruses characterized by high genetic diversity, a high rate of nucleotide substitutions in the genome and frequent genomic recombination. These factors make these viruses responsible for zoonotic infections in humans starting from animal reservoirs through “cross-species infections” by intermediate hosts [5]. Coronaviruses have a single-stranded RNA genome with positive polarity that is covered by an envelope. SARS-CoV-2 has an RNA genome containing nucleotides, coding for 9860 amino acids. Phylogenetic analysis by genomic sequencing showed high similarity between SARS-CoV-2 and bat coronaviruses, Bat-CoVRaTG13 (96.3%), Bat-SL-CoVZC45 and Bat-SL-CoVZXC21 (88%) [6], but minor similarity with SARS-CoV (79%) and MERS-CoV (50%) [7].

The genome consists of six major coding regions (open reading frames, ORFs), ORF1a/b, S, E, M, N and other accessory genes. The ORF1a/b region encodes a polyprotein replicase. The S gene encodes the spike glycoprotein, which is involved in binding to the ACE2 cell receptor. This glycoprotein consists of two domains, S1 and S2, which mediate adhesion to the receptor and entry of the virus into the cell, respectively. The receptor-binding domain (RBD) is located in the C-terminal region of the S1 protein, which binds the angiotensin-converting enzyme (ACE2) cell receptor expressed by alveolar type II (AT2) pneumocytes, similar to SARS-CoV. This bond determines a conformational change in the protein, which implies the exposure of the S2 domain that mediates membrane fusion, allowing the entry of the virus into the lung cell. Finally, the genes E, M and N encode envelope, membrane and nucleocapsid, respectively [7]. In contrast, MERS-CoV binds a different cell receptor, i.e., dipeptidyl dipeptidase 4 (DPP4 or CD26), which is also expressed by various renal cells, justifying the infiltration of the virus and the consequent kidney damage [8].

In addition, SARS-CoV was able to infect cells of the immune system, such as macrophages and T cells; this feature is not yet known for SARS-CoV-2 [2].

### 2.1. Immune Response to Infection

The host immune response addresses viral infection through innate and acquired mechanisms. It has been hypothesized that the innate immune response mechanism to SARS-CoV-2 is analogous to that against other RNA viruses. Viral antigens behave as pathogen-associated molecular patterns (PAMPs) capable of binding TLRs (TLR3 and TLR7) and cytosolic RNA receptors. This event leads to the activation of the intracellular NF-kB pathway, which induces the activation of the IFN-I gene, stimulating the JAK-STAT pathway and finally activating the IFN-stimulated response elements (ISREs) responsible for the suppression of viral replication and the dissemination of the virus in the early stages of infection [2].

SARS-CoV and MERS-CoV can suppress the innate immune response through multiple interference strategies with intracellular signaling pathways. Due to its high similarities with these viruses, it is hypothesized that SARS-CoV-2 also uses these strategies to modulate the immune response in the host. However, in some severe respiratory manifestations caused by SARS-CoV and MERS-CoV, an excessive innate immune response has been observed, with dysregulation of IFN-I activity and a negative impact on the outcome of the infection. Furthermore, for SARS-CoV-2, transmission of the virus by asymptomatic individuals has been documented, probably due to a delay in the innate immune response [2].

In contrast, the adaptive immune response is manifested by cellular Th1 type activation, which determines a high production of proinflammatory cytokines (IL-2, IL-6, IL-10, G-CSF, interferon-inducible protein [IP] 10, TNF-α and others), called a “cytokine storm”. The adaptive immune response seems to be fundamental to the control of SARS-CoV, MERS-CoV and SARS-CoV-2 infections. All this evidence justifies the laboratory pattern of neutrophilia and lymphopenia associated with an increase in the serological values of IL-6 and C reactive protein (CRP) commonly found in infected patients. Although the cytotoxic cellular T response is essential for infected cell elimination, it appears to be involved in the genesis of lung damage [2].

The humoral response leads to the production of neutralizing antibodies against the main viral epitopes (the proteins S, M, E, and N), which are responsible for limiting infection and preventing future infections. SARS-CoV induces seroconversion within 4 days from the start of the infection, and specific protective IgG dosages have been found up to 2 years after the infection. MERS-CoV induces seroconversion within 2–3 weeks from the start of the infection. Preliminary data on SARS-CoV suggest that IgM-IgG switching occurs within the first two weeks of infection. The serum of five COVID-19 patients showed cross-reactivity with SARS-CoV [2].

The production of a vaccine against SARS-CoV-2 is necessary to control and reduce the transmission of the virus, creating herd immunity [9]. Vaccines bring about a reduction in the so-called “R0 value”, or the “number of basic reproductions”, which represents the average number of secondary infections produced by each infected individual in a completely susceptible population, i.e., those that have never come into contact with the new emerging pathogen. This parameter measures the potential transmissibility of an infectious disease. According to the WHO, in the first stage of the epidemic, 2019-nCoV had an estimated R0 between 1.4 and 3.8 [10].

### 2.2. Epitopes of SARS-CoV-2

A complete knowledge of the main viral antigens is important to produce an effective vaccine. A study by Baruah and Bose describes the results from amino acid sequences of the main antigens of SARS-CoV-2 [11]. They compared them with those of SARS-CoV and other coronaviruses using immunoinformatic techniques to identify effective B and T cell epitopes. The authors highlighted that the 2019-nCoV surface glycoprotein (S) has an identity of 76.3% and a similarity of 87.3% with the “spike glycoprotein” of SARS-CoV [11]. Specifically, they identified five MHC-I-binding epitopes capable of inducing an effective immune response and three continuous and five discontinuous B cell epitopes. With the exception of a single CTL epitope (VVNQNAQAL), which showed 100% identity with that of SARS-CoV, the other epitopes shared only partial identity with those of SARS-CoV, MERS-CoV and bat-CoV [11].

According to Han et al., the S1 and S2 domains of the 2019-nCoV spike glycoprotein are, respectively, approximately 70% and 99% similar to those of SARS-CoV [7]. The viral envelope protein (E) represents another target for potential vaccines. In particular, studies on SARS-CoV and MERS-CoV mutants lacking the E protein showed that they were able to replicate (replicant competent) but were defective in propagation (propagation defective) [8,12].

## 3. Types of Vaccines

SARS-CoV-2 vaccines will be essential to reducing morbidity and mortality if the virus establishes itself in the population [13]. Genetic engineering techniques have greatly expanded in recent decades, allowing the production of increasingly effective and safe vaccines, although they can be traced back to the classic vaccine families, which we will summarize below.

Viral vector-based vaccines are produced by grafting the genome of a vector virus with a portion of viral DNA coding for immunogenic components, whose expression leads to the activation of an effective cellular and humoral immune response [14]. In contrast, DNA vaccines are composed of a recombinant plasmid encoding viral immunogens, which are expressed by infected host cells and able to elicit humoral and cellular responses. They are inexpensive and easy to produce. These vaccines can induce a T cellular response and a robust humoral immune response, but spontaneous plasmid integration into host genomes represents a potential risk, although the probability is extremely low [15,16].

Subunit vaccines are developed based on synthetic peptides or recombinant proteins. These vaccines generally present a high safety profile but low immunogenicity [14].

Inactivated whole-virus vaccines (IWVs) represent the historical type of vaccine produced by chemical or thermal inactivation of complete viruses. These are inexpensive and safe vaccines because they do not involve genetic manipulation. However, their production requires high levels of containment and has the disadvantage of altering or reducing the immunogenicity of the main viral epitopes during the inactivation phases.

The main feature of live-attenuated vaccines is the capability to induce immunity similar to that induced by natural infection. Generally, their production involves the deletion of viral genes that confer virulence, they do not require adjuvants for high immunogenicity, and they display optimal efficacy to evoke a robust immune response after a single immunization. Nevertheless, live-attenuated vaccines are associated with the risk of unwanted adverse effects, such as reversion to a virulent strain and opportunistic infections, which prevent administration in immunocompromised and elderly people [14].

Finally, virus-like particle (VLP)-based vaccines are based on nanoscale particles similar to native viral particles without infectious genetic materials, so they are non-replicative and non-infectious. The VLP-based vaccine is similar to the whole-virus inactivated vaccine, but its production does not require a high-containment structure because no live virus is involved in the manufacturing process [14].

In 2002–2003, the world experienced the first lethal coronavirus infection, and the disease denominated severe acute respiratory syndrome (SARS) has been characterized by high fever, eventually developing into shortness of breath and pneumonia [17]. Originating in southern China, 8096 cases resulted in 774 deaths in 26 countries [18]. Despite efforts from the scientific community, no vaccine became commercially available, and SARS cases ceased to be reported in 2004 [17].

Reasons for the lack of commercial vaccines for SARS-CoV are varied, but the main point seems to be the low interest in investing in a disease whose cases ceased to be reported in 2004 and that has produced relatively few and geographically centralized cases (compared with those of HIV, influenza and tuberculosis).

### 3.1. Natural Infection and Protection

Exposure to SARS-CoV can also guide possible mechanisms of protection. Studies in humans have reported that rapid and strong neutralizing antibody responses are highly correlated with the severity of the disease [19,20,21]. From these studies, it has been noted that the induction of both responses, T cells and antibodies, is necessary for the effective elimination of the virus and recovery from the disease. The long-term protection from vaccination or exposure to SARS-CoV is under debate, but memory T cells but not B cells could be detected 6 years after infection in human survivors [22].

A certain level of antibodies can be tracked until one year after infection, opening the possibility of a certain level of protection during this time due to this humoral response [23]. Studies have shown that neutralizing antibodies against SARS-CoV S glycoproteins play a predominant role in protection; indeed, vaccinated animals and passive immunization approaches focusing on coronavirus glycoproteins induced high titers of antibodies that correlated with protection [24]. It is in doubt that adaptative T cell responses can also play a role in conferring protection. There are a few studies focused on this problem; thus, a group of researchers demonstrated that specific CD4+ and CD8+ T cells against SARS-CoV could be exploited to provide protection in mice [25,26,27]. Another study showed that the protection against SARS-CoV in mice induced by a DNA vaccine was due only to antibody responses; indeed, the depletion of CD4 CD8 T cells and adaptive T cell transfer did not have an effect on protection [28].

Few animal experiments have tried to explain whether a certain vaccination regimen could induce long-term protection. Data on the use of viral vectors and protein-based vaccines employing the S glycoprotein have shown a certain level of protection from infection 4–12 months after vaccination in at least 75% of mice [29]. However, there are several difficulties in building up adequate animal models (i.e., small animal models and non-human primate models) capable of recapitulating disease clinical signs in humans [30].

### 3.2. SARS-CoV Immunological Studies

The initial phase of vaccine development against SARS-CoV-2 could consider the high genetic similarity between SARS-CoV-2 and SARS-CoV. Studies have shown that by screening SARS-CoV-derived B cell and T cell epitopes in immunogenic structural proteins, there are a set of B cell and T cell epitopes that map identically to the SARS-CoV-2 proteins. As no mutation has been observed in these identified epitopes among the 120 available SARS-CoV-2 sequences (as of 21 February 2020), immune targeting of these epitopes may potentially offer protection against this novel virus (Syed F-A Preliminary Identification of 2020). On the T cell side, the identification of SARS-CoV-derived epitopes that map identically to SARS-CoV-2 and the large population that these are expected to cover are encouraging. This finding promotes further research in exploring vaccines designed to induce a protective T cell response, which has been shown to provide long-term protection against SARS-CoV [22,31,32].

### 3.3. Vaccines for SARS-CoV

Several vaccines for SARS-CoV were developed and tested in animal models, including recombinant S-protein-based vaccines, vectored vaccines and inactivated whole-virus vaccines [33]. Most of these vaccines protect animals from challenge with SARS-CoV, but many do not induce sterilizing immunity.

The majority of the subunit vaccines targeted the S spike glycoprotein of the virus because it uses this protein to bind and enter host cells; thus, a vaccine that induces strong immune responses against this protein will have a significant effect on the deterrence of virus entry into host cells during natural infection. Many preclinical studies have been performed: vaccines have been based on a live-attenuated or inactivated virus, recombinant viral vectors, DNA, virus-like particles (VLPs) and soluble proteins.

### 3.4. Animal Models for Vaccines

The results showed complete protection in mouse models after two doses (as measured by viral loads in the lungs), complete protection in rhesus macaques after two doses as measured by viral shedding, and no evidence of adverse effects after challenge due to vaccination. Live attenuated/host adapted SARS virus (E-deleted) vaccines were tested only in animal models in preclinical studies; mice showed the induction of neutralizing antibodies and CD4/CD8 responses, without side effects [34,35,36,37,38,39,40,41]. In the case of recombinant viral vectors, viruses other than SARS-CoV that are capable of host cell infection have been genetically engineered to express components of SARS-CoV. The recombinant modified vaccinia Ankara (MVA) virus was used in a preclinical trial that demonstrated induction of neutralizing antibodies from SARS-CoV replication in the lungs in mice and induction of protection from virus shedding and viral replication in macaques, although protection was not observed in ferrets. This trial demonstrated side effects, such as high levels of ALT, indicating hepatic lesions when expressing the S protein and hepatitis after challenge with SARS-CoV in ferrets [24,42,43,44].

A recombinant non-replicating adenovirus (E-deleted) vaccine used in a preclinical trial in an animal model demonstrated superior cellular immune responses in the lungs of mice after intranasal and sublingual immunizations than after intramuscular immunization, but a side effect was the redirection of the vectors to the olfactory bulbs by intranasal administration [45,46,47].

VLP adjuvants are non-infectious multiprotein structures formed from viral proteins that self-assemble into virus-like structures [48]. For VLPs, a preclinical study in mice resulted in the induction of neutralizing antibodies and protection from SARS-CoV replication in the lungs, but after this challenge, there was evidence of a degree of lung immunopathology [49].

Table 1 shows SARS-CoV vaccine candidates in clinical trials in animals.

### 3.5. Human Vaccine Clinical Trials

Only a small number of vaccines against SARS-CoV progressed to phase I clinical trials before funding expired because of the natural eradication of the virus from the human population through non-pharmaceutical interventions when case numbers were still small. Only vaccines based on an inactivated SARS-CoV, DNA and soluble proteins based on the SARS-CoV S glycoprotein reached a clinical stage (phase I).

In live-attenuated and inactivated viruses using whole SARS-CoV as a vaccine, the virus was rendered non-replicating, and infectivity was reduced by deleting components of the virus genome or by using chemical or physical methods [34].

The results of the inactivated SARS-CoV vaccine (preclinical studies and phase I clinical trial) were as follows: in humans, there was induction of significant titers of neutralizing antibodies after two immunizations (100% seroconversion in participants), and the vaccine was well tolerated with no severe adverse effects (mild adverse effects such as local pain, erythema, abdominal pain or diarrhea) that resolved in 24 h. In particular, 4 out of 32 individuals reported reverted high levels of ALT at a certain dose [35,36,37]. DNA-based vaccines reached a clinical stage (phase I clinical trial), and neutralizing antibodies were detected after 2–3 doses in 8/10 human subjects and T cell responses in 10/10 subjects. In humans, there were no side effects. In animal models, neutralizing antibodies and CD4+/CD8+ T cell responses were induced in mice, but protection from viral replication was dependent on humoral responses and not T cell responses [38].

It is necessary for the development of vaccines to determine whether they can provide protection from viral infection. This determination is usually achieved by exposing vaccinated individuals and model animals to the virus in question. Due to the virulence of SARS-CoV, challenge studies in humans were not performed, and thus, the protective efficacy of the vaccines was not assessed.

Data suggest that SARS-CoV vaccines might cross-protect against SARS-CoV-2 because some neutralizing monoclonal antibodies isolated against SARS-CoV, such as CR3022, can cross-react with the receptor-binding domain of SARS-CoV-2 [39].

However, because these vaccines have not been developed further than phase I, they are currently not available for use.

Table 2 reports SARS-CoV vaccine candidates in human clinical trials.

#### Safety Issues

In some cases, vaccination with live virus results in complications, including infiltration of eosinophils and lung damage in a mouse model [36,49] and liver damage in ferrets [43]. Another study identified certain epitopes on the S protein as protective, whereas immunity to others seemed to enhance disease; however, vaccination is associated with greater survival, reduced virus titer and less morbidity compared with those in unvaccinated animals [50].

There is a concern about the induction of antibody-dependent enhancement (ADE) and other adverse effects with coronavirus vaccination. ADE is the enhancement of virus infectivity that occurs when non-neutralizing antibodies against proteins of a virus enhance virus entry to host cells [51]. ADE has already been observed in cats vaccinated against a species-specific coronavirus; however, the induction of ADE using the S glycoprotein has been approached by using only a domain of these proteins. Indeed, it is believed that the use of the S1 subunit of the S glycoprotein or the receptor-binding domain (RBD) can lead to the induction of neutralizing antibodies while avoiding ADE. The goal is to focus the induction of antibodies to relevant S regions for efficient virus neutralization and to avoid the induction of potential non-neutralizing antibodies targeting other regions of the S protein [52].

The use of adjuvants has also been considered for avoiding the potential adverse effect of coronavirus vaccination because these are substances that potentiate and increase the immunogenicity and protection efficacy of vaccines [53]. A study by Honda-Okubo et al. found that by using a chemical adjuvant, such as a delta inulin-based polysaccharide, lung immunopathology previously observed in mice after SARS-CoV challenge experiments was no longer observed [54]. It was hypothesized that the adjuvant helped to avoid an exacerbated Th2 response after challenge that causes the adverse effects.

## 4. MERS-CoV

The MERS-CoV epidemic began in September 2012 in the Middle East, where it spread, causing over 400 infections and 130 deaths in two years. The high lethality of the virus (approximately 35%) led to the engagement of strategies that make diagnosis and treatment earlier to limit the incidence of new infections over the years. In 2019, approximately 2500 cases and over 800 deaths were certified by the WHO, some of which were attributable to second- and third-generation contacts in South Korea [14]. Although the contagions are meagre, MERS-CoV remains a socio-health problem to date, so the need to find a vaccine pushes research forward. In 2019, Yong et al. carried out a thorough review of the literature on potential vaccines against MERS-CoV. Most of the potential vaccines studied are in the preclinical phase, and only three vaccine candidates, GLS-5300, MERS001 and MVA-MERS-S, entered human clinical trials with adult volunteer candidates [4,14].

### 4.1. Viral Vector-Based Vaccine

Three of these kinds of vaccines were produced through recombinant human adenoviral vectors (type 5 or 41) encoding the complete S protein. Kim et al., based on previous studies, identified an adenoviral vaccine (rAd5) expressing the S1 subunit of the viral S protein capable of inducing the production of neutralizing antibodies in mice [55]. Similarly, Guo et al. evaluated the antigen-specific immune response, both mucosal and systemic, induced in vivo by a single intramuscular or intragastric administration of a recombinant vaccine. This construct was produced by adenoviral vectors type 5 or 41 (Ad5 or Ad41) expressing the S protein. Both vaccines were able to induce high production of RBD-specific IgG four weeks after administration. The serum titer of antibodies induced by the Ad41-based vaccine was five times higher than that induced by the Ad5-based vaccine. Moreover, the intramuscolar (i.m.) route was more effective than the intragastric (i.g.) route because it induced both a T cellular and humoral response, with higher serum nAb levels [56]. Later, Hashem et al. studied an intramuscularly administered vaccine produced by the fusion of a viral gene encoding the S1-CD40L protein with a vector genome (rAd5). Mice immunized with two doses of this vaccine developed higher levels of nAbs against MERS-CoV after a single dose than those immunized with vaccines produced without CD40L. Therefore, they highlighted the adjuvant function of CD40L to increase the immunogenicity of vaccines based on the S1 protein [57].

Other recombinant vaccines have exploited a replication-defective adenoviral vector of monkeys (ChAd), which shows a good safety and immunogenicity profile in humans. In this way, Munster et al. produced a ChAdOx1-MERS vaccine using the viral S glycoprotein gene inserted in the E1 region of the ChAdOx1 vector. This construct was able to evoke a robust humoral and T CD8+ cellular response in lethal BALB/c transgenic mice expressing the gene for the human DPP4 receptor (hDPP4). Alharbi et al. obtained similar results, producing a ChAdOx1 vaccine with human tissue plasminogen activator (tPA) [58].

The MERS001 vaccine, which consists of the ChAdOx1 plasmid encoding the MERS-CoV S protein, is currently in the clinical trial phase. Forty-eight volunteer adult participants were enrolled for this study, and the outcome is assessing the safety profile and immunogenicity of this vaccine. The volunteers were divided into five study groups to which the vaccine was administered with different methods and dosages, according to a protocol [59].

Other potential recombinant vaccines have been produced using the viral vector MVA (modified vaccinia virus Ankara). Three of these presented the viral genome grafted with the viral S protein gene [60,61,62] and one with the N protein gene [62]. The MVA-MERS-S vaccine candidate belongs to this group. It is in phase I of human clinical trials in Germany, where a monocentre, non-randomized study was conducted to assess its safety and immunogenicity profile on a voluntary adult population of twenty-eight participants, according to a protocol of two increasing doses [63].

Finally, the vaccine produced by Jung et al. (2018) appears innovative, consisting of adenoviral vectors and protein nanoparticles of S protein, which was able to induce an effective Th1 and Th2 cellular response in transgenic mice [64].

### 4.2. DNA Vaccine

GLS-5300 belongs to this family of vaccines and is one of three vaccines in phase I/II clinical trials.

This vaccine was produced from the work of Muthumani et al., who synthesized a plasmid (pVax1) containing gene sequences coding for the different domains of the S protein associated with a sequence coding for a high efficiency immunoglobulin E leader peptide to facilitate the expression and export of RNA [15]. Subsequently, the vaccine was administered in mice, camels and rhesus macaques to evaluate immunization. After administration in mice, polyclonal activation of T lymphocytes was noted. Cytometry studies on cytokines produced allowed identification of cell clones: these were mainly TNFα-secreting CD4+ cells and IFNγ-producing CD8+ cells. In addition, serological analysis in vaccinated mice revealed a robust humoral immune response against the S protein, and the antibody titer increased after each vaccine administration. Immunized camels and macaques also developed a robust immune response [15]. In September 2019, Modjarrad et al. published a phase I clinical trial on the GLS-5300 vaccine, enrolling a population of 67 volunteers and using a dose-escalation protocol. The GLS-5300 vaccine contained 6 mg/mL pGX9101 plasmid, containing a gene insert of the MERS-CoV S protein. The different dosages (0.67 mg, 2 mg, and 6 mg) were administered by i.m. injection into the deltoid following electroporation to increase the entry of the DNA plasmid into cells. The side effects, adverse reactions, safety profile and host response (immunogenicity) were studied. In particular, the ability to induce a T cell response was assessed by measuring IFNγ with ELISPOT (IFNγ-ELISPOT) and determining the anti-protein S1 antibody titer. Forty-four participants developed a T cell response after the third vaccination (week 14), and forty-two maintained it until the end of the study (week 60). Fifty-nine participants developed anti-MERS-CoV S1 protein antibodies after the third vaccination, and fifty-two of them maintained seroreactivity until the end of the study (week 60). In addition, the authors highlighted a dose-independent response to plasmid concentrations in the vaccine [65].

A phase I/II study currently ongoing in South Korea could provide additional information on this vaccine.

Table 3 describes MERS-CoV vaccine candidates in human clinical trials.

### 4.3. Subunit Vaccine

The main targets of these vaccines are the S1 subunit and RBD of the S protein. Although subunit vaccines based on the full-length S protein may elicit robust immune responses, many studies have found that some of these vaccines could mediate an enhancement of viral infection in vitro [66].

Regarding S1 domain subunit vaccines, Wang et al. tested the immune response to the S377-588-Fc vaccine candidate in hDPP4 transgenic mice. This vaccine was produced using the S1 protein associated with adjuvants, such as alum and MF59, and administered subcutaneously. The authors highlighted that two doses of the vaccine 4 weeks apart induced serum nAbs and Th1 and Th2 cellular responses greater than a single administration or with boosters 1–2 weeks apart and are positively associated with protection against MERS-CoV [67].

Similarly, Adney et al. published an experimental study on a potential subunit vaccine containing the S1 protein produced by a vector encoding a codon-optimized S1 gene. The authors tested the effects of the potential vaccine on animal reservoirs (camels) and surrogate animal models (alpacas) by administering 400 µg of S1 protein combined with 40 mg of HCXL adjuvant in two intramuscular administrations 28 days apart, followed by administration of 400 µg of S1 protein with Sigma Adjuvant System at day 105. This construct was able to reduce and delay viral shedding in the upper respiratory tract of dromedary camels and induced complete protection in alpacas against infection by MERS-CoV [68].

To date, most studies have reported encouraging data on the use of the RBD to produce potential vaccines.

One of the first studies was that of Du et al., which identified the ability of the RBD region of the Spike glycoprotein to elicit the production of neutralizing antibodies [62,69]. Similar results were also obtained by Mou et al. [70]. Therefore, in a subsequent study, they identified a truncated fragment of the RBD containing residues 377–588, including the receptor-binding motif (RBM) region. They produced a fusion protein combining this fragment with the Fc region of human antibodies to increase its immunogenicity and stability in vivo [69]. This product, named S377-588-Fc, was able to induce the production of neutralizing antibodies, especially in the acute phase of the disease and in the presence of an adjuvant (Montanide ISA 51) [71].

In 2014, Ma et al. tested the ability of recombinant RBD fragments containing MERS-CoV residues 358–588 and 377–662 to elicit a neutralizing antibody response in mice and rabbits. Among the fragments evaluated, S377-588-Fc demonstrated high receptor affinity and the ability to elicit the highest antibody titer in mice and a high titer in rabbits [72]. A subsequent study by Ma et al. highlighted how intranasal (i.n.) administration of the same type of vaccine could induce an immune response similar to that induced by the subcutaneous (s.c.) route, especially with a predominance of the Th1 cell response associated with IgG2a in vaccinated mice. Furthermore, i.n. administration induced a robust mucosal antibody response represented by secretory IgA, which protected vaccinated laboratory mice from MERS-CoV infection more strongly than in subcutaneous administration [73].

Other studies evaluated the efficacy and safety of potential RBD-based subunit vaccines associated with different adjuvants. Tests on transgenic mice showed that they were fully protected against MERS-CoV infection and had no major morbidity, indicating the efficacy and safety of the candidate vaccines [74].

In particular, Lan et al. carried out some studies on potential vaccines constructed using the RBD motif of the spike protein combined with adjuvants, such as Freund’s adjuvant (IFA), alum, cytosine-phosphate-guanine (CpG), and polyriboinosinic acid (poly (I: C)), because of their ability to activate immunity through toll-like receptors (TLRs) and to polarize the T cell response. In 2014, the group produced a recombinant form of the RBD (rRBD) using the baculovirus expression system. The potential vaccine was then administered to BALB/c transgenic mice, with three i.m. or s.c. injections 3 weeks apart. Serological analysis of the mice showed that the vaccine produced in combination with the IFA and CpG adjuvants was able to elicit a robust antibody response similar to that produced by the combination with alum and CpG but induced a lower production of neutralizing antibodies. In addition, the RBD associated with alum and CpG stimulated a mixed T cell response, with a prevalence of Th1 cells [75].

In a later study, Lang et al. evaluated rRBD subunit vaccines in non-human primate animal models (rhesus macaques) to test their efficiency in infectious prophylaxis. The vaccine produced through the baculovirus expression vector system was mixed with an adjuvant, alum, one day before administration. The results showed that serological IgG antibody titers increased significantly after the first vaccination, reaching the maximum titer 2 weeks after the second immunization. Neutralizing antibodies appeared only after the second immunization. In addition, animals infected with MERS-CoV developed less serious clinical pictures and a lower incidence of serious pneumonia, although organ injuries at autopsies were not fully absent. The viral load found in lung, trachea and oropharynx swabs of immunized macaques decreased [76].

### 4.4. Inactivated Whole-Virus Vaccine (IWV)

One of the first studies on these potential MERS-CoV vaccines was that of Agrawal et al. [77]. It was prepared by irradiating MERS-CoV from a culture of Vero-E6 cells with γ-rays and then subjected to centrifugation and analysis by Western blot, which demonstrated the presence of viral structural proteins such as the S protein and N nucleoprotein. Then, this vaccine was administered in the presence of one or more adjuvants (alum or MF59) to BALB/c mice, which were divided into study groups according to protocol, with two i.m. injections 3 weeks apart. Serological studies 21 days following the second immunization showed the presence of high antibody titers in mice vaccinated with adjuvants. Microbiological analysis by PCR in infected mice showed a reduced viral load. However, some mice presented lung lesions with peribronchiolar or perivascular eosinophilic infiltrates attributable to hypersensitivity reactions. This condition was confirmed by the finding of elevated levels of Th2 cytokines, such as IL-5 and IL-13 [77].

Additionally, Wirblich et al. published a study on an inactivated whole-virus vaccine produced through a viral vector (BNSP333) derived from an attenuated Rabdovirus expressing MERS-CoV S1. This chimeric RABV vaccine was then tested on hDPP4+ transgenic mice. Serological analysis revealed a high titer of neutralizing antibodies against MERS-CoV [78].

Finally, in 2018, Deng et al. published a study to evaluate the effects of an inactivated whole MERS-CoV (IV) or S protein vaccine combined with an adjuvant (alum + CpG). The authors immunized transgenic BALB/c mice and evaluated the effects on MERS-CoV challenge. Serum neutralizing antibody production was reported, but no cell-mediated immune response occurred [79].

### 4.5. Live-Attenuated Vaccine

In 2013, Almazan et al. produced a live-attenuated vaccine of a MERS-CoV mutant using a full-length plasmid, deleting the E gene but preserving the M gene. This mutant was able to replicate but was infection defective [12]. A study by Menachery et al. on attenuated MERS-CoV mutants defective for the dNSP16 protein showed that these strains did not present effective IFN activation and post-infection viral replication in vitro and in vivo. Based on previous studies, the authors tested the ability of this potential vaccine to induce protection against MERS-CoV infection in transgenic mice. Immunized mice had less viral replication and a lower incidence of pulmonary hemorrhages than controls [80].

Other studies on this type of vaccine highlighted the use of replication-competent recombinant measles virus (MV) expressing foreign antigens. In 2015, Malczyk et al. produced an attenuated but replication-competent recombinant, MV, expressing the S gene of MERS-CoV. The virus was able to produce a truncated form of the S protein lacking the transmembrane domain. The immunogenicity was subsequently evaluated in IFNAR/CD46Ge transgenic mice. The serum of the mice displayed neutralizing antibodies directed both against MV proteins and against the S protein of MERS-CoV. Furthermore, the mice were protected against virus challenge [81]. Additionally, Bodmer et al. produced a potential vaccine using a recombinant MV but expressing the MERS-CoV N protein (MVvac2-MERS-N). In accordance with the aim of the study, they highlighted the vaccine’s ability to induce a significant antigen-specific T cell response in transgenic mice [82]. Finally, Liu et al. constructed a VSV-based recombinant chimeric virus encoding MERS-CoV S-protein as a membrane glycoprotein instead of its own G protein. The authors demonstrated that single-dose immunization by either the i.m. or i.n. route induced high-level and lasting MERS-CoV nAbs and T cell responses in rhesus monkeys. Quantitative ELISA results showed that the recombinant virus induced higher levels of S protein-specific IgG via the i.m. route than via the i.n. route [83].

### 4.6. Virus-Like Particle (VLP)-Based Vaccine

Wang et al. constructed a recombinant baculovirus co-expressing the S, E and M genes of MERS-CoV and then evaluated its immunogenicity as a vaccine candidate in rhesus macaques. After immunization with alum adjuvant, rhesus macaques developed nAbs and a high titer of IgG against the RBD of MERS-CoV. Moreover, this vaccine elicited a Th1-mediated response based on the detection of IFN-gamma [84].

A similar study by Coleman et al. described a recombinant baculovirus produced using the full-length S genes of SARS-CoV and MERS-CoV. The authors highlighted that mice vaccinated with coronavirus S nanoparticles produced high levels of neutralizing antibodies against homologous virus but did not offer cross-protection against the heterologous virus. Moreover, it emerged that the use of adjuvants boosts the production of neutralizing antibodies [85]. Later, Coleman et al. tested the capability of that vaccine candidate to protect mice from MERS-CoV challenge. They immunized BALB/c hDPP+ mice in the presence of an adjuvant (Matrix-M1TM) at days 0 and 21 and then evaluated sera by ELISA. Transgenic mice were divided into study subgroups and immunized according to a protocol and then infected with MERS-CoV. Sera from mice vaccinated with the Matrix-M1 adjuvant showed significantly higher neutralizing antibody titers than PBS-vaccinated controls. Moreover, virus replication was inhibited in the lungs of vaccinated mice [86].

A novel type of VLP-based vaccine was produced by Wang et al. using a chimeric canine parvovirus (CPV) VLP expressing the RBD of MERS-CoV. They tested immunogenicity in 32 BALB/c mice randomized into four groups, which were vaccinated i.m. with different adjuvants (alum or poly (I: C)) or PBS. ELISA revealed an RBD-specific antibody response at two weeks after the first injection, which was stronger after the second immunization and in the presence of the poly (I: C) adjuvant [87].

In contrast, Lan et al. produced a novel type of cVLP using a baculovirus insect cell expression system containing a modified MERS-CoV S protein and avian influenza matrix 1 (M1). They assessed the immunogenicity of the vaccine associated with different types of adjuvants (alum or CpG) in BALB/c mice. After the third immunization, they presented the highest IgG titer and highest neutralizing antibody titer [88].

Table 4 reports MERS-CoV vaccine candidates in pre-clinical trials in animals.

## 5. SARS-CoV-2

On 11 April 2020, the WHO published a summary of the main vaccine candidates against COVID-19 in pharmacological experimentation. These are over 120 potential vaccines, of which over 70 are in the preclinical phase and 7 are in the clinical trial phase [89].

A recombinant SARS-CoV-2 vaccine based on an adenovirus vector is currently in phase II clinical trials in China. It is a randomized, double blinded, placebo-controlled study in healthy adults aged above 18 years, whose aim is to evaluate the safety profile, anti-S IgG and anti-SARS-CoV-2 neutralizing antibodies at 6 months after vaccination [90].

A phase I/II clinical trial of a viral vector-based vaccine candidate (ChAdOx1 n-CoV-19) is ongoing in the UK. It is a single-blind randomized clinical trial on 1112 adult volunteer participants aimed at assessing its safety, efficacy and immunogenicity profile. The study protocol provides for the division of the participants into seven groups (Groups 1a, 1b, 2a, 2b, 3, 4a, and 4b). Groups 1a, 2a, 3, and 4a were administered the ChAdOx1 vaccine by the i.m. route, while the remaining control groups were immunized with the MenACWY vaccine [91].

In the US, a phase I non-randomized clinical trial is underway on a DNA vaccine candidate (INO-4800). The study evaluated the safety, tolerability and immunogenicity profile of a population of 40 adult volunteer participants distributed into two study groups: the first group was administered 1 mg of INO-4800 i.m., followed by electroporation (EP); the second group was administered two doses of 1 mg of INO-4800 i.m. 4 weeks apart, and each one was followed by EP [92].

Two other studies were performed on inactivated virus vaccines. One of these is ongoing in China, and it is a randomized, double-blind, placebo parallel-controlled phase I/II clinical trial for an inactivated novel coronavirus pneumonia vaccine (from Vero cells). The study evaluated the safety profile of the vaccine in a healthy population of different ages, inoculating different doses of the COVID-19 vaccine (from Vero cells), and exploring its immunogenicity and efficacy [93].

Additionally, in China, a second study is underway on an inactivated vaccine for SARS-CoV-2. It is a randomized, double-blind, parallel-controlled placebo phase I/II clinical trial of a SARS-CoV-2 inactivated vaccine tested on an adult population of 18–59 years old. Different vaccine dosages are administered to participants according to a protocol. The primary objectives of the study are the evaluation of the safety profile and immunogenicity indexes of neutralizing antibody seroconversion rates [94].

In Germany, a multi-site phase I/II clinical trial is underway. It is a dose-escalation trial investigating the safety and immunogenicity of four potential RNA vaccines (BNT162a1, BNT162b1, BNT162b2, and BNT162c2) against SARS-CoV-2 through a protocol with different dosages in healthy adults. Seroconversion was defined as a minimum fourfold increase in antibody titer from baseline [95].

Finally, the last clinical trial is an American study on a potential mRNA-1273 vaccine. It is a phase I, open-label, dose-ranging clinical trial in males and non-pregnant females who are in good health and meet all eligibility criteria. The aim of the trial is to evaluate the safety, reactogenicity and immunogenicity of the mRNA-1273 vaccine. mRNA-1273 is a novel lipid nanoparticle (LNP)-encapsulated mRNA-based vaccine that encodes a full-length, prefusion stabilized spike protein (S) of SARS-CoV-2. Forty-five participants were enrolled into three different cohorts (25 mcg, 100 mcg, 250 mcg) administered two i.m. injections in the deltoid muscle two weeks apart. They will be followed through 12 months post-second vaccination (day 394) to evaluate any adverse reactions and to measure the antibody titer developed by ELISA. Seroconversion is defined as a fourfold change in antibody titer from baseline [96].

Table 5 describes SARS-CoV-2 vaccine candidates in human clinical trials.

To date, however, marketing of an effective vaccine remains a distant goal, probably the first quarter of 2021 due to several time frame problems. First, the vaccine is tested in appropriate animal models to determine whether it is protective. Second, vaccines need to be tested for toxicity in animals. This testing, which has to be performed in a manner compliant with Good Laboratory Practice (GLP), typically takes 3–6 months to complete. If there are already sufficient data available for similar vaccines made in the same production process, these safety tests might be skipped. Once sufficient pre-clinical trial data are available and there are initial batches of the vaccine with good manufacturing practice (GMP) quality, clinical trials might be initiated. Development of vaccines starts with small phase I trials to evaluate the safety in humans; followed by phase II trials, in which formulation and doses are established to initially prove efficacy; and finally, by phase III trials in which the efficacy and safety of a vaccine need to be assessed in a larger cohort population. In an extraordinary situation such as the current one, this scheme might be compressed, and an accelerated regulatory approval pathway might be developed. If efficacy is demonstrated, a vaccine might be licensed by regulatory agencies.

An important point is that the production capacity to produce enough GMP-quality vaccine needs to be available. For vaccines based on existing vaccine platforms (inactivated or live-attenuated vaccines), this can be relatively easily achieved, while for vaccines based on novel technologies (mRNA), this capacity needs to be built, and this development takes time. Finally, it takes time to distribute vaccines and administer them. Given that the population is currently naive to SARS-CoV-2, it is likely that more than one dose is necessary, and usually two vaccinations are spaced 3–4 weeks apart. It is likely that protective immunity will be achieved only 1–2 weeks after the second vaccination. According to that information, it is unlikely that a vaccine would be available earlier than 6 months after the initiation of clinical trial.

## 6. Conclusions

Despite the numerous vaccines undergoing clinical and preclinical studies, the long trial times slow down the fight against coronaviruses. This extensive review carried out on the vaccine candidates of the main epidemic coronaviruses of the past has shown that the studies in animal models suggest a high efficacy of potential vaccines in providing protection against viral challenges. Similar human studies have not yet been carried out, as the main trials are aimed at assessing mainly vaccine safety and immunogenicity. Whereas the SARS-CoV epidemic ended almost two decades ago and the MERS-CoV epidemic is now better controlled, as it is less contagious due to the high lethality of the virus, the current SARS-CoV-2 pandemic represents a problem that is certainly more compelling, which pushes us to accelerate the studies not only for the production of vaccines but also for innovative pharmacological treatments. SARS-CoV-2 vaccines might come too late to affect the first wave of this pandemic, but they might be useful if additional subsequent waves occur or in a post-pandemic perspective in which the virus continues to circulate as a seasonal virus.

## Figures and Tables

**Table 1:** Severe acute respiratory syndrome (SARS-CoV) vaccine candidates in clinical trials in animals.

Vaccine	Target	Outcome	Side Effects	Status	References
Live-attenuated/host-adapted SARS-CoV (E-deleted)	Whole genome except the envelope	Mice: Induction of neutralizing antibodies and CD4/CD8+ T cell responses	Not reported	Pre-clinical	[39,40]
Recombinant modified vaccinia Ankara virus	Spike or N protein	Mice: induction of neutralizing antibodies and protection from SARS-CoV replication in the lungs Rhesus macaques: induction of protection from virus shedding and viral replication in the lungs Ferrets: not protective against SARS-CoV replication and shedding	High level of ALT, indicating hepatic lesions when expressing the S protein and hepatitis after challenge with SARS-CoV in ferrets	Pre-clinical	[23,41,42,43]
Recombinant non-replicating adenovirus (E-deleted)	Spike or N protein	Mice: superior cellular immune responses in the lung after intranasal and sublingual immunization than after intramuscular immunization Mice and ferrets: reduction in virus replication and shedding	Mice: redirection of the vector to the olfactory bulbs by intranasal administration	Pre-clinical	[44,45,46]
VLPs	Spike	Mice: induction of neutralizing antibodies and protection from SARS-CoV replication in the lungs	Evidence of a degree of lung immunopathology in mouse models	Pre-clinical	[47,48]

**Table 2:** SARS-CoV vaccine candidates in human clinical trials.

Vaccine	Target	Outcome	Side Effects	Status	References
Inactivated SARS-CoV	All structural proteins	Humans: induction of significant titers of neutralizing antibodies after two immunizations (100% of 32 individuals)	Few cases of mild side effects that resolved in 24 h (local pain, erythema, diarrhea)	Phase I	[34,35,36]
DNA-based vaccines	Full spike S glycoprotein or fragment	Humans: induction of neutralizing antibodies (8/10) and T cell responses (10/10) after 2–3 doses	Well tolerated	Phase I	[37]

**Table 3:** Middle East respiratory syndrome (MERS-CoV) vaccine candidates in human clinical trials.

Vaccine	Type	Target	Vector/Adjuvant	Stage	References
MERS001	Viral vector based	S protein	ChAdOx1	Phase I	[58]
MVA-MERS-S	Viral vector based	S protein	MVA	Phase I	[62]
GLS-5300	DNA vaccine	S protein	pGX9101	Phase I/II	[64]

**Table 4:** MERS-CoV vaccine candidates in pre-clinical trials in animals.

Vaccine	Target	Vector/Adjuvant	Outcome	References
**Viral vector based**	S1 subunit/RBD	Human adenovirus (Ad)	Strong humoral and cellular responses	[54,55,56]
	S protein	Monkey adenovirus (ChAd)	Humoral and T CD8+ responses	[57]
	S protein N proteins	Modified vaccinia Ankara virus (MVA)	nAbs ± CD8+ responses	[59,60,61]
	S protein	Adenovirus + protein nanoparticles	Th1 and Th2 cellular responses	[63]
**DNA vaccine**	S protein	Plasmid pVax1	Polyclonal T lymphocyte activation and robust immune response, increasing after each administration	[12]
**Subunit vaccine**	S1 subunit	Alum, MF59, HCXL, Sigma Adjuvant System	nAbs + cellular responses provide protection against virus challenge in animal models	[66,67]
	RBD fused to Fc	Montanide ISA 51, others	nAbs	[70,71,72]
	Recombinant RBD	IFA, alum, CpG, poly(I: C)	Low nAb levels, robust T cellular response, protection against virus challenge and decreased viral load in animal models	[73,74,75]
**Inactivated whole-virus (IWV)**	S protein, N nucleoprotein	Alum, MF59	Robust humoral response	[76,77,78]
		BNSP333		
		Alum + CpG		
**Live-attenuated**	S protein	MERS-CoV mutant E deleted	Infection-defective virus	[11]
		MERS-CoV mutant dNSP16 deleted	Induced protection against MERS-CoV and less lung lesions in mice	[79]
	Truncated S protein	Recombinant measles virus (MV)	nAbs and protection against virus challenge	[80]
	N protein		T cellular response in mice	[81]
	S protein + G protein (VSV)	Recombinant VSV	nAbs + T cellular responses in macaques	[82]
**VLPs**	S, E, M protein	Baculovirus	Humoral + Th1 cellular responses	[83]
	S protein	Baculovirus	Humoral response, induced protection in mice	[84,85]
	RBD	Chimeric canine parvovirus (CPV)	Humoral response	[86]
	S protein + M1 (Influenza)	Alum/CpG	Humoral response	[87]

**Table 5:** SARS-CoV-2 vaccine candidates in human clinical trials.

Vaccine	Target	Vector/Adjuvant	Type of Study	Stage	Participants	Country	References
Viral vector based	S protein	Adenovirus vector	Randomized, double-blinded	Phase II	500	China	[89]
Viral vector based (ChAdOx1 n-CoV-19)	S protein	Canine adenovirus vector	Randomized, single-blinded	Phase I/II	1112	UK	[90]
DNA vaccine (INO-4800)	n.e.	Electroporation	Non-randomized	Phase I	40	USA	[91]
Inactivated whole-virus	n.e.	n.e.	Randomized, double-blinded	Phase I/II	288 (I), 1168 (II)	China	[92]
Inactivated whole-virus	n.e.	n.e.	Randomized, double-blinded	Phase I/II	744	China	[93]
RNA vaccines (BNT162a1, BNT162b1 BNT162b2 and BNT162c2)	n.e.	n.e.	Non-randomized	Phase I/II	196	Germany	[94]
LNP-encapsulated mRNA-vaccine (mRNA-1273)	S protein	Lipid nanoparticles	Non-randomized	Phase I	45	USA	[95]

## References

[1] Shanmugaraj B., Malla A., Phoolcharoen W. (2020). Emergence of Novel Coronavirus 2019-nCoV: Need for Rapid Vaccine and Biologics Development. Pathogens.

[2] Prompetchara E., Ketloy C., Palaga T. (2020). Immune responses in COVID-19 and potential vaccines: Lessons learned from SARS and MERS epidemic. Asian Pac. J. Allergy Immunol..

[3] Zhang L., Liu Y. (2020). Potential interventions for novel coronavirus in China: A systematic review. J. Med. Virol..

[4] Xu J., Jia W., Wang P., Zhang S., Shi X., Wang X., Zhang L. (2019). Antibodies and vaccines against Middle East respiratory syndrome coronavirus. Emerg. Microbes Infect..

[5] She J., Jiang J., Ye L., Hu L., Bai C., Song Y. (2020). 2019 novel coronavirus of pneumonia in Wuhan, China: Emerging attack and management strategies. Clin. Transl. Med..

[6] Lu R., Zhao X., Li J., Niu P., Yang B., Wu H., Wang W., Song H., Huang B., Zhu N. (2020). Genomic characterisation and epidemiology of 2019 novel coronavirus: Implications for virus origins and receptor binding. Lancet.

[7] Han Q., Lin Q., Jin S., You L. (2020). Coronavirus 2019-nCoV: A brief perspective from the front line. J. Infect..

[8] Ralph R., Lew J., Zeng T., Francis M., Xue B., Roux M., Ostadgavahi A.T., Rubino S., Dawe N.J., Al-Ahdal M.N. (2020). 2019-nCoV (Wuhan virus), a novel Coronavirus: Human-to-human transmission, travel-related cases, and vaccine readiness. J. Infect. Dev. Ctries..

[9] Pang J., Wang M.X., Ang I.Y.H., Tan S.H.X., Lewis R.F., Chen J.I.-P., A Gutierrez R., Gwee S.X.W., Chua P.E.Y., Yang Q. (2020). Potential Rapid Diagnostics, Vaccine and Therapeutics for 2019 Novel Coronavirus (2019-nCoV): A Systematic Review. J. Clin. Med..

[10] Italian Higher Institute of Health. https://www.iss.it/primo-piano/-asset_publisher/o4oGR9qmvUz9/content/id/526885.

[11] Baruah V., Bose S. (2020). Immunoinformatics-aided identification of T cell and B cell epitopes in the surface glycoprotein of 2019-nCoV. J. Med. Virol..

[12] Almazán F., Dediego M.L., Sola I., Zuñiga S., Nieto-torres J.L., Marquez-jurado S., Andrés G. (2013). A Vaccine Candidate East Respiratory Syndrome Coronavirus as a Vaccine Candidate. MBio.

[13] Amanat F., Krammer F. (2020). SARS-CoV-2 Vaccines: Status Report. Immunity.

[14] Muthumani K., Falzarano D., Reuschel E.L., Tingey C., Flingai S., Villarreal D.O., Wise M., Patel A., Izmirly A., Aljuaid A. (2015). A synthetic consensus anti–spike protein DNA vaccine induces protective immunity against Middle East respiratory syndrome coronavirus in nonhuman primates. Sci. Transl. Med..

[15] Chi H., Zheng X., Wang X., Wang C., Wang H., Gai W., Perlman S., Yang S., Zhao J., Xia X. (2017). DNA vaccine encoding Middle East respiratory syndrome coronavirus S1 protein induces protective immune responses in mice. Vaccine.

[16] Yong C.Y., Ong H., Yeap S.K., Ho K.L., Tan W. (2019). Recent Advances in the Vaccine Development Against Middle East Respiratory Syndrome-Coronavirus. Front. Microbiol..

[17] NHS SARS: Severe Acute Respiratory Syndrome. https://www.nhs.uk/conditions/sars/.

[18] World Health Organization Summary of Probable SARS Cases with Onset of Illness from 1 November 2002 to 31 July 2003 Based on Data as of the 31 December 2003. http://www.who.int/csr/sars/country/table2004_04_21/en/index.

[19] Li T., Xie J., He Y., Fan H., Baril L., Qiu Z., Han Y., Xu W., Zhang W., You H. (2006). Long-Term Persistence of Robust Antibody and Cytotoxic T Cell Responses in Recovered Patients Infected with SARS Coronavirus. PLoS ONE.

[20] Park W.B., Perera R.A.P.M., Choe P.G., Lau E.H., Choi S.J., Chun J.Y., Oh H.S., Song K.-H., Bang J.H., Kim E.S. (2015). Kinetics of Serologic Responses to MERS Coronavirus Infection in Humans, South Korea. Emerg. Infect. Dis..

[21] Gu J., Gong E., Zhang B., Zheng J., Gao Z., Zhong Y., Zou W., Zhan J., Wang S., Xie Z. (2005). Multiple organ infection and the pathogenesis of SARS. J. Exp. Med..

[22] Tang F., Quan Y., Xin Z., Wrammert J., Ma M.-J., Lv H., Wang T.-B., Yang H., Richardus J.H., Liu W. (2011). Lack of Peripheral Memory B Cell Responses in Recovered Patients with Severe Acute Respiratory Syndrome: A Six-Year Follow-Up Study. J. Immunol..

[23] Liu W., Fontanet A., Zhang P., Zhan L., Xin Z., Baril L., Tang F., Lv H., Cao W.-C. (2006). Two-Year Prospective Study of the Humoral Immune Response of Patients with Severe Acute Respiratory Syndrome. J. Infect. Dis..

[24] Bisht H., Roberts A., Vogel L., Bukreyev A., Collins P.L., Murphy B.R., Subbarao K., Moss B. (2004). Severe acute respiratory syndrome coronavirus spike protein expressed by attenuated vaccinia virus protectively immunizes mice. Proc. Natl. Acad. Sci. USA.

[25] Zhao J., Zhao J., Mangalam A.K., Channappanavar R., Fett C., Meyerholz D.K., Agnihothram S., Baric R.S., David C.S., Perlman S. (2016). Airway Memory CD4(+) T Cells Mediate Protective Immunity against Emerging Respiratory Coronaviruses. Immunity.

[26] Liu W.J., Lan J., Liu K., Deng Y., Yao Y., Wu S., Chen H., Bao L., Zhang H., Zhao M. (2016). Protective T Cell Responses Featured by Concordant Recognition of Middle East Respiratory Syndrome Coronavirus–Derived CD8+ T Cell Epitopes and Host MHC. J. Immunol..

[27] Zhao J., Zhao J., Perlman S. (2010). T Cell Responses Are Required for Protection from Clinical Disease and for Virus Clearance in Severe Acute Respiratory Syndrome Coronavirus-Infected Mice. J. Virol..

[28] Yang Z.-Y., Kong W.-P., Huang Y., Roberts A., Murphy B.R., Subbarao K., Nabel G.J. (2004). A DNA vaccine induces SARS coronavirus neutralization and protective immunity in mice. Nature.

[29] Du L., Zhao G., He Y., Guo Y., Zheng B.-J., Jiang S., Zhou Y. (2007). Receptor-binding domain of SARS-CoV spike protein induces long-term protective immunity in an animal model. Vaccine.

[30] Totura A., Bavari S. (2019). Broad-spectrum coronavirus antiviral drug discovery. Expert Opin. Drug Discov..

[31] Fan Y.-Y., Huang Z.-T., Li L., Wu M.-H., Yu T., Koup R.A., Bailer R.T., Wu C.-Y. (2009). Characterization of SARS-CoV-specific memory T cells from recovered individuals 4 years after infection. Arch. Virol..

[32] Ng O.-W., Chia A., Tan A.T., Jadi R.S., Leong H.N., Bertoletti A., Tan Y.-J. (2016). Memory T cell responses targeting the SARS coronavirus persist up to 11 years post-infection. Vaccine.

[33] Roper R.L., E Rehm K. (2009). SARS vaccines: Where are we?. Expert Rev. Vaccines.

[34] Petrovsky N., Aguilar J.C. (2004). Vaccine adjuvants: Current state and future trends. Immunol. Cell Boil..

[35] Lin J., Zhang J.-S., Su N., Xu J., Wang N., Chen J.-T., Chen X., Liu Y.-X., Gao H., Jia Y.-P. (2007). Safety and immunogenicity from a phase I trial of inactivated severe acute respiratory syndrome coronavirus vaccine. Antivir. Ther..

[36] Bolles M., E Deming M., Long K., Agnihothram S., Whitmore A., Ferris M., Funkhouser W., Gralinski L., Totura A., Heise M. (2011). A Double-Inactivated Severe Acute Respiratory Syndrome Coronavirus Vaccine Provides Incomplete Protection in Mice and Induces Increased Eosinophilic Proinflammatory Pulmonary Response upon Challenge. J. Virol..

[37] Orellana C. (2004). Phase I SARS vaccine trial in China. Lancet Infect. Dis..

[38] Martin J.E., Louder M.K., Holman L.A., Gordon I.J., Enama M.E., Larkin B.D., Andrews C.A., Vogel L., Koup R.A., Roederer M. (2008). A SARS DNA vaccine induces neutralizing antibody and cellular immune responses in healthy adults in a Phase I clinical trial. Vaccine.

[39] Tian X., Li C., Huang A., Xia S., Lu S., Shi Z., Lu L., Jiang S., Yang Z., Wu Y. (2020). Potent binding of 2019 novel coronavirus spike protein by a SARS coronavirus-specific human monoclonal antibody. Emerg. Microbes Infect..

[40] Regla-Nava J.A., Nieto-Torres J.L., Jimenez-Guardeño J.M., Fernández-Delgado R., Fett C., Castaño-Rodriguez C., Perlman S., Enjuanes L., DeDiego M.L. (2015). Severe Acute Respiratory Syndrome Coronaviruses with Mutations in the E Protein Are Attenuated and Promising Vaccine Candidates. J. Virol..

[41] Fett C., DeDiego M.L., Regla-Nava J.A., Enjuanes L., Perlman S. (2013). Complete Protection against Severe Acute Respiratory Syndrome Coronavirus-Mediated Lethal Respiratory Disease in Aged Mice by Immunization with a Mouse-Adapted Virus Lacking E Protein. J. Virol..

[42] Czub M., Weingartl H., Czub S., He R., Cao J. (2005). Evaluation of modified vaccinia virus Ankara based recombinant SARS vaccine in ferrets. Vaccine.

[43] Weingartl H., Czub M., Czub S., Neufeld J., Marszal P., Gren J., Smith G., Jones S., Proulx R., Deschambault Y. (2004). Immunization with Modified Vaccinia Virus Ankara-Based Recombinant Vaccine against Severe Acute Respiratory Syndrome Is Associated with Enhanced Hepatitis in Ferrets. J. Virol..

[44] Chen Z., Zhang L., Qin C., Ba L., Yi C.E., Zhang F., Wei Q., He T., Yu W., Yu J. (2005). Recombinant Modified Vaccinia Virus Ankara Expressing the Spike Glycoprotein of Severe Acute Respiratory Syndrome Coronavirus Induces Protective Neutralizing Antibodies Primarily Targeting the Receptor Binding Region. J. Virol..

[45] Shim B.-S., Stadler K., Nguyen H.H., Yun C.-H., Kim D.W., Chang J., Czerkinsky C., Song M.K. (2012). Sublingual immunization with recombinant adenovirus encoding SARS-CoV spike protein induces systemic and mucosal immunity without redirection of the virus to the brain. Virol. J..

[46] See R.H., Zakhartchouk A.N., Petric M., Lawrence D.J., Mok C.P.Y., Hogan R.J., Rowe T., Zitzow L.A., Karunakaran K.P., Hitt M.M. (2006). Comparative evaluation of two severe acute respiratory syndrome (SARS) vaccine candidates in mice challenged with SARS coronavirus. J. Gen. Virol..

[47] See R.H., Petric M., Lawrence D.J., Mok C.P.Y., Rowe T., Zitzow L.A., Karunakaran K.P., Voss T.G., Brunham R.C., Gauldie J. (2008). Severe acute respiratory syndrome vaccine efficacy in ferrets: Whole killed virus and adenovirus-vectored vaccines. J. Gen. Virol..

[48] Hamilton K., Melia T., Monni P.F., Re E., Zanderighi G. (2016). Merging W W and W W + jet with Minlo. J. High Energy Phys..

[49] Tseng C.-T., Sbrana E., Iwata-Yoshikawa N., Newman P.C., Garron T., Atmar R.L., Peters C.J., Couch R.B. (2012). Immunization with SARS coronavirus vaccines leads to pulmonary immunopathology on challenge with the SARS virus. PLoS ONE.

[50] Wang Q., Zhang L., Kuwahara K., Li L., Liu Z., Li T., Zhu H., Liu J., Xu Y., Xie J. (2016). Immunodominant SARS Coronavirus Epitopes in Humans Elicited both Enhancing and Neutralizing Effects on Infection in Non-human Primates. ACS Infect. Dis..

[51] Dimmock N.J., Easton A.J., Leppard K.N. (2016). Introduction to Modern Virology.

[52] Du L., Zhao G., Lin Y., Chan C.C., He Y., Jiang S., Wu C., Jin N.-Y., Yuen K.-Y., Zhou Y. (2008). Priming with rAAV encoding RBD of SARS-CoV S protein and boosting with RBD-specific peptides for T cell epitopes elevated humoral and cellular immune responses against SARS-CoV infection. Vaccine.

[53] Hearnden C., Lavelle E.C. (2013). Adjuvant Strategies for Vaccines: The Use of Adjuvants within the Cancer Vaccine Setting. Cancer Immunotherapy.

[54] Honda-Okubo Y., Barnard D., Ong C.H., Peng B.-H., Tseng C.-T.K., Petrovsky N. (2014). Severe Acute Respiratory Syndrome-Associated Coronavirus Vaccines Formulated with Delta Inulin Adjuvants Provide Enhanced Protection while Ameliorating Lung Eosinophilic Immunopathology. J. Virol..

[55] Kim E., Okada K., Kenniston T., Raj V.S., Alhajri M.M., Farag E.A., Alhajri F., Osterhaus A., Haagmans B.L., Gambotto A. (2014). Immunogenicity of an adenoviral-based Middle East Respiratory Syndrome coronavirus vaccine in BALB/c mice. Vaccine.

[56] Guo X., Deng Y., Chen H., Lan J., Wang W., Zou X., Hung T., Lu Z.-Z., Tan W. (2015). Systemic and mucosal immunity in mice elicited by a single immunization with human adenovirus type 5 or 41 vector-based vaccines carrying the spike protein of Middle East respiratory syndrome coronavirus. Immunology.

[57] Hashem A.M., Algaissi A., Agrawal A.S., Al-Amri S.S., Alhabbab R.Y., Sohrab S.S., Almasoud A.S., Alharbi N.K., Peng B.-H., Russell M. (2019). A Highly Immunogenic, Protective, and Safe Adenovirus-Based Vaccine Expressing Middle East Respiratory Syndrome Coronavirus S1-CD40L Fusion Protein in a Transgenic Human Dipeptidyl Peptidase 4 Mouse Model. J. Infect. Dis..

[58] Munster V., Wells D., Lambe T., Wright D., Fischer R.J., Bushmaker T., Saturday G., Van Doremalen N., Gilbert S.C., De Wit E. (2017). Protective efficacy of a novel simian adenovirus vaccine against lethal MERS-CoV challenge in a transgenic human DPP4 mouse model. NPJ Vaccines.

[59] National Institutes of Health Safety and Immunogenicity of a Candidate MERS-CoV Vaccine (MERS001). https://clinicaltrials.gov/ct2/show/study/NCT03399578.

[60] Alharbi N.K., Padron-Regalado E., Thompson C., Kupke A., Wells D., Sloan M.A., Grehan K., Temperton N.J., Lambe T., Warimwe G. (2017). ChAdOx1 and MVA based vaccine candidates against MERS-CoV elicit neutralising antibodies and cellular immune responses in mice. Vaccine.

[61] Volz A., Kupke A., Song F., Jany S., Fux R., Shams-Eldin H., Schmidt J., Becker C., Eickmann M., Becker S. (2015). Protective Efficacy of Recombinant Modified Vaccinia Virus Ankara Delivering Middle East Respiratory Syndrome Coronavirus Spike Glycoprotein. J. Virol..

[62] Veit S., Jany S., Fux R., Sutter G., Volz A. (2018). CD8+ T Cells Responding to the Middle East Respiratory Syndrome Coronavirus Nucleocapsid Protein Delivered by Vaccinia Virus MVA in Mice. Viruses.

[63] National Institutes of Health Safety, Tolerability and Immunogenicity of Vaccine Candidate MVA-MERS-S. https://clinicaltrials.gov/ct2/show/NCT03615911.

[64] Jung S.-Y., Kang K.W., Lee E.Y., Seo D.-W., Kim H.-L., Kim H., Kwon T., Park H.-L., Kim H., Lee S.-M. (2018). Heterologous prime-boost vaccination with adenoviral vector and protein nanoparticles induces both Th1 and Th2 responses against Middle East respiratory syndrome coronavirus. Vaccine.

[65] Modjarrad K., Roberts C.C., Mills K.T., Castellano A.R., Paolino K., Muthumani K., Reuschel E.L., Robb M.L., Racine T., Oh M.-D. (2019). Safety and immunogenicity of an anti-Middle East respiratory syndrome coronavirus DNA vaccine: A phase 1, open-label, single-arm, dose-escalation trial. Lancet Infect. Dis..

[66] Wang N., Shang J., Jiang S., Du L. (2020). Subunit Vaccines against Emerging Pathogenic Human Coronaviruses. Front. Microbiol..

[67] Wang Y., Tai W., Yang J., Zhao G., Sun S., Tseng C.-T.K., Jiang S., Zhou Y., Du L., Gao J. (2017). Receptor-binding domain of MERS-CoV with optimal immunogen dosage and immunization interval protects human transgenic mice from MERS-CoV infection. Hum. Vaccines Immunother.

[68] Adney D.R., Wang L., Van Doremalen N., Shi W., Zhang Y., Kong W.-P., Miller M.R., Bushmaker T., Scott D., De Wit E. (2019). Efficacy of an Adjuvanted Middle East Respiratory Syndrome Coronavirus Spike Protein Vaccine in Dromedary Camels and Alpacas. Viruses.

[69] Du L., Zhao G., Kou Z., Ma C., Sun S., Poon V.K.M., Lu L., Wang L., Debnath A.K., Zheng B.-J. (2013). Identification of a Receptor-Binding Domain in the S Protein of the Novel Human Coronavirus Middle East Respiratory Syndrome Coronavirus as an Essential Target for Vaccine Development. J. Virol..

[70] Mou H., Raj V.S., Van Kuppeveld F.J.M., Rottier P.J.M., Haagmans B.L., Bosch B.-J. (2013). The Receptor Binding Domain of the New Middle East Respiratory Syndrome Coronavirus Maps to a 231-Residue Region in the Spike Protein That Efficiently Elicits Neutralizing Antibodies. J. Virol..

[71] Du L., Kou Z., Ma C., Tao X., Wang L., Zhao G., Chen Y., Yu F., Tseng C.-T.K., Zhou Y. (2013). A Truncated Receptor-Binding Domain of MERS-CoV Spike Protein Potently Inhibits MERS-CoV Infection and Induces Strong Neutralizing Antibody Responses: Implication for Developing Therapeutics and Vaccines. PLoS ONE.

[72] Ma C., Wang L., Tao X., Zhang N., Yang Y., Tseng C.-T.K., Li F., Zhou Y., Jiang S., Du L. (2014). Searching for an ideal vaccine candidate among different MERS coronavirus receptor-binding fragments—The importance of immunofocusing in subunit vaccine design. Vaccine.

[73] Ma C., Li Y., Wang L., Zhao G., Tao X., Tseng C.-T.K., Zhou Y., Du L., Jiang S. (2014). Intranasal vaccination with recombinant receptor-binding domain of MERS-CoV spike protein induces much stronger local mucosal immune responses than subcutaneous immunization: Implication for designing novel mucosal MERS vaccines. Vaccine.

[74] Nyon M.P., Du L., Tseng C.-T.K., Seid C., Pollet J., Naceanceno K.S., Agrawal A., Algaissi A., Peng B.-H., Tai W. (2018). Engineering a stable CHO cell line for the expression of a MERS-coronavirus vaccine antigen. Vaccine.

[75] Lan J., Deng Y., Chen H., Lu G., Wang W., Guo X., Lu Z.-Z., Gao G.F., Tan W. (2014). Tailoring Subunit Vaccine Immunity with Adjuvant Combinations and Delivery Routes Using the Middle East Respiratory Coronavirus (MERS-CoV) Receptor-Binding Domain as an Antigen. PLoS ONE.

[76] Lan J., Yao Y., Deng Y., Chen H., Lu G., Wang W., Bao L., Deng W., Wei Q., Gao G.F. (2015). Recombinant Receptor Binding Domain Protein Induces Partial Protective Immunity in Rhesus Macaques Against Middle East Respiratory Syndrome Coronavirus Challenge. EBioMedicine.

[77] Agrawal A.S., Tao X., Algaissi A., Garron T., Narayanan K., Peng B.-H., Couch R.B., Tseng C.-T. (2016). Immunization with inactivated Middle East Respiratory Syndrome coronavirus vaccine leads to lung immunopathology on challenge with live virus. Hum. Vaccines Immunother..

[78] Wirblich C., Coleman C., Kurup D., Abraham T.S., Bernbaum J.G., Jahrling P.B., Hensley L.E., Johnson R.F., Frieman M.B., Schnell M.J. (2016). One-Health: A Safe, Efficient, Dual-Use Vaccine for Humans and Animals against Middle East Respiratory Syndrome Coronavirus and Rabies Virus. J. Virol..

[79] Deng Y., Lan J., Bao L., Huang B., Ye F., Chen Y., Yao Y., Wang W., Qin C., Tan W. (2018). Enhanced protection in mice induced by immunization with inactivated whole viruses compare to spike protein of middle east respiratory syndrome coronavirus. Emerg. Microbes Infect..

[80] Menachery V.D., Gralinski L.E., Mitchell H.D., Dinnon K.H., Leist S.R., Yount B.L., Graham R.L., McAnarney E.T., Stratton K.G., Cockrell A.S. (2017). Middle East Respiratory Syndrome Coronavirus Nonstructural Protein 16 Is Necessary for Interferon Resistance and Viral Pathogenesis. mSphere.

[81] Malczyk A.H., Kupke A., Prüfer S., Scheuplein V.A., Hutzler S., Kreuz D., Beissert T., Bauer S., Hubich-Rau S., Tondera C. (2015). A Highly Immunogenic and Protective Middle East Respiratory Syndrome Coronavirus Vaccine Based on a Recombinant Measles Virus Vaccine Platform. J. Virol..

[82] Bodmer B.S., Fiedler A.H., Hanauer J.R., Prüfer S., Mühlebach M.D. (2018). Live-attenuated bivalent measles virus-derived vaccines targeting Middle East respiratory syndrome coronavirus induce robust and multifunctional T cell responses against both viruses in an appropriate mouse model. Virology.

[83] Liu R., Wang J., Shao Y., Wang X., Zhang H., Shuai L., Ge J., Wen Z., Bu Z. (2017). A recombinant VSV-vectored MERS-CoV vaccine induces neutralizing antibody and T cell responses in rhesus monkeys after single dose immunization. Antivir. Res..

[84] Wang C., Zheng X., Gai W., Zhao Y., Wang H., Wang H., Feng N., Chi H., Qiu B., Li N. (2016). MERS-CoV virus-like particles produced in insect cells induce specific humoural and cellular imminity in rhesus macaques. Oncotarget.

[85] Coleman C., Liu Y.V., Mu H., Taylor J.K., Massare M., Flyer D.C., Glenn G.M., Smith G.E., Frieman M.B. (2014). Purified coronavirus spike protein nanoparticles induce coronavirus neutralizing antibodies in mice. Vaccine.

[86] Coleman C., Venkataraman T., Liu Y.V., Glenn G.M., Smith G.E., Flyer D.C., Frieman M.B. (2017). MERS-CoV spike nanoparticles protect mice from MERS-CoV infection. Vaccine.

[87] Wang C., Zheng X., Gai W., Wong G., Wang H., Jin H., Feng N., Zhao Y., Zhang W., Li N. (2017). Novel chimeric virus-like particles vaccine displaying MERS-CoV receptor-binding domain induce specific humoral and cellular immune response in mice. Antivir. Res..

[88] Lan J., Deng Y., Song J., Huang B., Wang W., Tan W. (2018). Significant Spike-Specific IgG and Neutralizing Antibodies in Mice Induced by a Novel Chimeric Virus-Like Particle Vaccine Candidate for Middle East Respiratory Syndrome Coronavirus. Virol. Sin..

[89] World Health Organization (2020). Draft of the Landscape of COVID-19 Candidate Vaccines.

[90] Insitute of Biotechnology, Academy of Military Medical Sciences, P. of C. A Randomized, Double-Blinded, Placebo-Controlled Phase II Clinical Trial for Recombinant Novel Coronavirus (2019-nCOV) Vaccine (Adenovirus Vector). http://www.chictr.org.cn/showprojen.aspx?proj=52006.

[91] National Institutes of Health A Study of a Candidate COVID-19 Vaccine (COV001). https://clinicaltrials.gov/ct2/show/NCT04324606.

[92] National Institutes of Health Safety, Tolerability and Immunogenicity of INO-4800 for COVID-19 in Healthy Volunteers. https://clinicaltrials.gov/ct2/show/NCT04336410.

[93] Wuhan Institute of Biological Products Evaluation of the Safety and Immunogenicity of Inactivated Novel Coronavirus Pneumonia (COVID-19) Vaccine (Vero cells) in Healthy Population Aged 6 Years and above: A Randomized, Double-Blind, Placebo Parallel-Controlled Phase I/II Clinical Trial. http://www.chictr.org.cn/showprojen.aspx?proj=52227.

[94] National Institutes of Health [NIH] Safety and Immunogenicity Study of Inactivated Vaccine for Prophylaxis of SARS CoV-2 Infection (COVID-19). https://clinicaltrials.gov/ct2/show/NCT04352608.

[95] European Union Clinical Trials Register Multi-site Phase I/II, 2-Part, Dose-Escalation Trial Investigating the Safety and Immunogenicity of four Prophylactic SARS-CoV-2 RNA Vaccines against COVID-2019 Using Different Dosing Regimens in Healthy Adults. https://www.clinicaltrialsregister.eu/ctr-search/search?query=BNT162-01.

[96] National Institutes of Health [NIH] Safety and Immunogenicity Study of 2019-nCoV Vaccine (mRNA-1273) for Prophylaxis SARS CoV-2 Infection (COVID-19). https://clinicaltrials.gov/ct2/show/NCT04283461.

